# Topologically Guided Prioritization of Candidate Gene Transcripts Coexpressed with the 5-HT_1A_ Receptor by Combining In Vivo PET and Allen Human Brain Atlas Data

**DOI:** 10.1093/cercor/bhz341

**Published:** 2020-01-16

**Authors:** Jakob Unterholzner, Gregor Gryglewski, Cecile Philippe, Rene Seiger, Verena Pichler, Godber M Godbersen, Neydher Berroterán-Infante, Matej Murgaš, Andreas Hahn, Wolfgang Wadsak, Markus Mitterhauser, Siegfried Kasper, Rupert Lanzenberger

**Affiliations:** 1 Department of Psychiatry and Psychotherapy, Medical University of Vienna, Währinger Gürtel 18-20, 1090, Vienna, Austria; 2 Division of Nuclear Medicine, Department of Biomedical Imaging and Image-guided Therapy, Medical University of Vienna, Währinger Gürtel 18-20, 1090, Vienna, Austria; 3 Centre for Biomarker Research in Medicine (CBmed), Stiftingtalstrasse 5, 8010, Graz, Austria; 4 Ludwig Boltzmann Institute Applied Diagnostics, Währinger Gürtel 18-20, 1090, Vienna, Austria

**Keywords:** coexpression, 5-HT1AR, mRNA, PET, Spearman

## Abstract

The serotonin-1A receptor (5-HT_1A_R) represents a viable target in the treatment of disorders of the brain. However, development of psychiatric drugs continues to be hindered by the relative inaccessibility of brain tissue. Although the efficacy of drugs selective for the 5-HT_1A_R has not been proven, research continues to focus on drugs that influence this receptor subtype. To further knowledge on this topic, we investigated the topological coexpression patterns of the 5-HT_1A_R. We calculated Spearman’s rho for the correlation of positron emission tomography-binding potentials (BP_ND_) of the 5-HT_1A_R assessed in 30 healthy subjects using the tracer [*carbonyl-*^11^C]WAY-100635 and predicted whole-brain mRNA expression of 18 686 genes. After applying a threshold of *r* > 0.3 in a leave-one-out cross-validation of the prediction of mRNA expression, genes with ρ ≥ 0.7 were considered to be relevant. In cortical regions, 199 genes showed high correlation with the BP_ND_ of the 5-HT_1A_R, in subcortical regions 194 genes. Using our approach, we could consolidate the role of *BDNF* and implicate new genes (*AnxA8*, *NeuroD2*) in serotonergic functioning. Despite its explorative nature, the analysis can be seen as a gene prioritization approach to reduce the number of genes potentially connected to 5-HT_1A_R functioning and guide future in vitro studies.

## Introduction

As the most widespread inhibitory serotonergic receptor (Kaufman et al. 2016), the serotonin-1A receptor (5-HT_1A_R) represents a viable target in the treatment of disorders of the brain. However, progress in the development of psychiatric drugs continues to be hindered by the relative inaccessibility of the brain, calling for new techniques to study disorder-relevant proteins and their interactions. More than 50 years have passed since the description of monoaminergic neurons of the central nervous system (Dahlstroem and Fuxe 1965) and the discovery of drug agents such as imipramine, reserpine, and monoaminoxidase inhibitors (Coppen 1967). In line with the monoaminergic hypothesis of depression (Schildkraut 1965), efficacy of, for example, selective serotonin reuptake inhibitors (SSRIs) has been shown repeatedly (Cipriani et al. 2018). Furthermore, drugs that block the 5-HT_1A_R seem to increase the effect of SSRIs (Artigas et al. 1996). Presynaptic 5-HT_1A_R desensitization or downregulation has been proposed as the underlying mechanism (Blier and Ward 2003). However, the efficacy of drugs selective for the 5-HT_1A_R in the treatment of disorders of the brain has not been proven as of yet. Still, research continues to focus on drugs that modulate this receptor subtype (Staroń et al. 2018). A more profound knowledge on the topological coexpression of the 5-HT_1A_R with other receptors and cellular proteins would certainly help in this regard.

The 5-HT_1A_R is a g-protein-coupled receptor and the most extensively studied receptor within the group of serotonergic receptors (Pucadyil et al. 2005). In the brain, the 5-HT_1A_R works as a presynaptic autoreceptor on serotonergic neurons in the dorsal and median raphe nuclei, whereas in the limbic system and the cortex, the 5-HT_1A_R predominantly functions as a postsynaptic receptor (Carhart-Harris and Nutt 2017). Altogether, receptor signaling is dependent of coupling between different receptors, and receptors and cellular proteins. Recent endeavors using biochemical and genetic assays have strived to better characterize g-protein-coupled receptors and their interacting proteins (Marin et al. 2012). While interesting, these rarely reflect the native cellular environment nor the topologic (co-)expression in the brain (Sokolina et al. 2017). The Allen Human Brain Atlas (AHBA), an extensive map of the mRNA transcriptome in the human adult brain derived from six healthy human brains (Hawrylycz et al. 2012), was a great development in this regard, allowing for the analysis of gene expression in different cell types and across different brain regions (Hawrylycz et al. 2012). Neuroimaging techniques, including positron emission tomography (PET), can offer additional in vivo information about the distribution of predefined molecules, for which radioactive tracers are available.

High correlation of protein distribution measured with PET and mRNA expression of the respective gene was shown for the 5-HT_1A_R (Komorowski et al. 2017), the metabotropic glutamate receptors 1 and 5 (Lohith et al. 2017), and the monoamine oxidase A (MAO-A) (Komorowski et al. 2017). In an effort to predict mRNA expression of 18 686 genes in the entire human brain, our group recently developed a method based on the microarray data from the AHBA and created whole-brain transcriptomic maps for each gene (Gryglewski et al. 2018).

In the current study, we present an explorative coexpression analysis of aforementioned predicted whole-brain mRNA expression of 18 686 genes and in-house PET-binding potential (BP_ND_) data for the 5-HT_1A_R using a Spearman correlation. This correlation only hints toward topological correlations between tracer binding and mRNA expression of diverse proteins and thus does not allow conclusions on functional or pathological protein–protein interactions. The present analysis can be seen as a gene prioritization approach to reduce the number of genes potentially involved in normal serotonergic functioning that can be tested in in vitro studies in the future and might be relevant as therapeutic targets.

## Materials and Methods

Correlation of mRNA expression intensity and nondisplaceable BP_ND_ for the 5-HT_1A_R is based upon a recently developed prediction model for unbiased whole-brain data (Gryglewski et al. 2018).

### PET and mRNA Datasets

PET BP_ND_ data were reanalyzed from a previous study (Lanzenberger et al. 2007). In this study, data were acquired from 30 healthy subjects (mean age 26.7 ± 6.8 years, 14 females) using a GE Advance PET scanner (Genera Electric Medical Systems, Milwaukee, Wisconsin) for 90 min after application of the selective tracer [carbonyl-^11^C]WAY-100635. T1-weighted structural data derived from an MPRAGE sequence on a Medspec 3T MRI scanner (Bruker BioSpin) were used for reconstruction of cortical surfaces in FreeSurfer 6.0. Quantification of BP_ND_ in Montreal Neurological Institute (MNI) space and on the cortical surface was performed using the multilinear reference tissue model (Ichise et al. 2003) with the insular cortex set as high-binding and cerebellar white matter as the reference region. The insular cortex was chosen because it represents a high-binding region with particularly stable kinetics and large spatial extent (Savli et al. 2012). This minimizes uncertainties in kinetic modeling and spatial normalization as well as influence from single outlier voxels. The study was approved by the ethics committee of the Medical University of Vienna and all subjects provided written informed consent.

Briefly, the prediction of expression patterns was based on Gaussian process regression (ordinary Kriging) implemented in the R package gstat 1.1–5 (Pebesma 2004). This entails the analysis of spatial dependence by means of variograms, which allow for the calculation of relative structured variability as a metric for the relative fraction of variance in gene expression between samples explained by their spatial distance. A high regional structured variability may be associated with low randomness or measurement error. The outcome of the prediction method was comprehensive datasets of each gene’s expression in three-dimensional MNI space and in FreeSurfer surface space. For a detailed description of the methodology, see Gryglewski et al. (2018).

### Coexpression Analysis

For each of the 18 686 genes, Spearman’s rho (ρ) was calculated to correlate its predicted mRNA expression intensity (to be found on http://www.meduniwien.ac.at/neuroimaging/mRNA.html) and both the BP_ND_ for the 5-HT_1A_R and the predicted mRNA expression intensity of the 5-HT1A-receptor. Correlations were calculated for the left hemisphere of the brain to avoid bias from the relative paucity of samples collected from the right hemisphere in the AHBA. Correlations were carried out vertex-wise in surface space for the cortex and voxel-wise in MNI space for subcortical regions. Genes with a ρ > 0.7 were considered to be highly coexpressed.

In a next step, a leave-one-out cross validation was performed by correlating observed and predicted mRNA expression for each gene using Pearson’s correlation coefficient. Genes with an *r* > 0.3 were selected in order to discard genes with low validity in prediction reflective of a low signal to noise ratio (Gryglewski et al. 2018).

To further prioritize genes coexpressed with the 5-HT_1A_R, the standard deviation (SD) of each gene across cortical and subcortical regions was used. The SD can be seen as a measure of the distribution of each gene. The SD for the BP_ND_ for the 5-HT_1A_R in cortical regions was calculated as SD = 0.44; in subcortical regions, it was calculated as SD = 2.31. Genes with an *r* > 0.3 and a ρ > 0.7 and an SD similar to that of the BP_ND_ of the 5-HT_1A_R, that is, the 90th percentile, were therefore selected to reflect coexpression with the 5-HT_1A_R.

**Figure 1:**
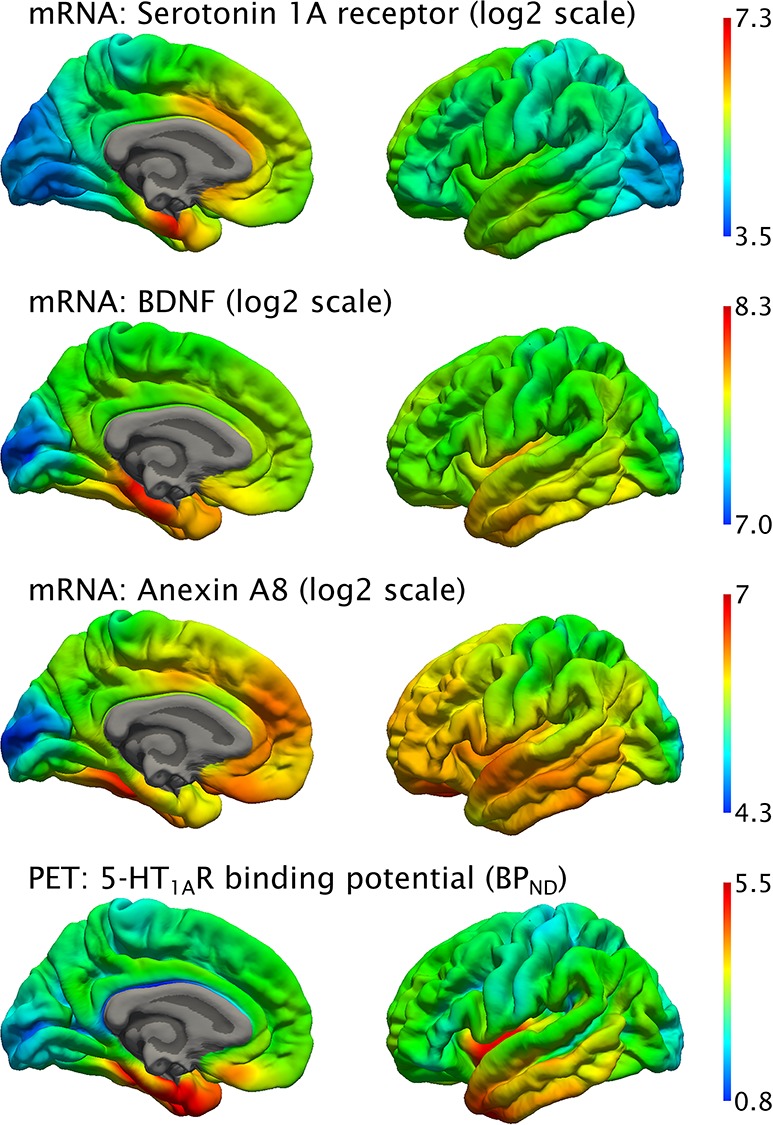
Predicted whole-brain mRNA expression of the *5-HT_1A_R*, *BDNF*, and *AnxA8* genes in the left cortical hemisphere (top three) and average BP_ND_ PET scans using [*carbonyl*-^11^C]WAY-100635 (bottom). mRNA expression intensity is given in units of log2. Left = medial view, right = lateral view.

### Gene Ontology Analysis

Protein Analysis Through Evolutionary Relationships (PANTHER) enable a functional classification of genes utilizing the gene ontology (GO) tool (Ashburner et al. 2000). For our GO analysis, we used the *GO complete* annotation set comprising about 45 000 distinct function terms (Mi et al. 2018). We performed an overrepresentation test for cellular components, molecular function, and biological processes using the PANTHER classification system analysis for the genes shown to be highly correlated with the BP_ND_ of the 5-HT_1A_R in cortical and subcortical regions, that is, *r* > 0.3 and ρ > 0.7 (Mi et al. 2019). This input list was compared to a reference list using a Fisher’s exact test and a false discovery rate (FDR) correction for multiple testing.

## Results

Vertex-wise Spearman’s rho and a consecutive leave-one-out analysis revealed 199 genes (i.e., predicted whole brain mRNA expression) correlated with the BP_ND_ of the 5-HT_1A_R measured with PET on the surface of the left hemisphere (ρ ≥ 0.7 and *r* > 0.3, supplementary tables S1 and S2). In subcortical regions, 194 genes were shown to be correlated with the BP_ND_ of the 5-HT_1A_R, when applying the methodology described above (also Supplementary Information). The predicted mRNA expression of the 5-HT_1A_R showed a strong correlation with the BP_ND_ of the 5-HT_1A_R in both cortical (ρ = 0.72) and subcortical (ρ = 0.84) regions. Apart from the 5-HT_1A_R gene, *AnxA8, BDNF, C4orf45, GPRIN1, HMGCR, KIAA1239, PGM2l1, PRSS2*, and *TNFAIP8L1* were shown to be correlated with the BP_ND_ of the 5-HT_1A_R in both cortical (Fig. 1) and subcortical regions.

All genes showing a similar SD (90th percentile) as the BP_ND_ of the 5-HT_1A_R, a ρ > 0.7 and an *r* > 0.3 can be found in Table 1.

In the GO analysis, genes highly correlated with the 5-HT_1A_R in cortical regions were found to be overrepresented in axons, the somatodendritic compartment and the plasma membrane bounded cell projection part of the data category cellular components (FDR corrected, *P* < 0.05; Table 2). For subcortical regions, the GO analysis revealed a significant overrepresentation of genes in the data categories of biological processes, that is, neuronal development, and cellular component, that is, in the axon, the presynapse and the cytoplasmic vesicle (also in Table 2).

## Discussion

In this exploratory analysis, we found 199 genes in cortical regions and 194 genes in subcortical regions associated with the 5-HT_1A_R using a correlation of predicted whole-brain mRNA expression of 18 686 genes and the BP_ND_ of the 5-HT_1A_R. With our in silico approach, we could reduce the number of genes potentially coexpressed with the 5-HT_1A_R to approximately 1% of the initial data set. When additionally including the SD of the BP_ND_ of the 5-HT_1A_R, only 0.1% of the genes remained. The mRNA of the 5-HT_1A_R was always among the 0.1–1% of genes shown to be highly correlated with the BP_NP_ of the 5-HT_1A_R, hinting toward a validity of our approach to define coexpression and prioritize genes with a shared function or intersecting secondary pathways.

The analysis at hand represents a correlation of the BP_ND_ of the tracer [*carbonyl*-^11^C]WAY-100635 with the whole-brain mRNA expression of the 18 686 genes. WAY-100635 binds the 5-HT_1A_R with high affinity and selectivity (Gozlan et al. 1995; Khawaja et al. 1995). Chemel et al. (2006) challenged the initial data of a 100-fold greater selectivity of WAY-100635 to bind the 5-HT_1A_R (Forster et al. 1995). In a follow-up study, Martel et al. (2007) could, however, show a 200-fold greater selectivity of WAY-100635 to bind the 5-HT_1A_R in comparison with, for example, the Dopamin-D4 receptor. Furthermore, the metabolism of [*carbonyl*-^11^C]WAY-100635 does not lead to radiolabelled WAY-100634, which would bind the Dopamin-D4 receptor. Also, based on the low expression of D4 receptor mRNA in the cortex, we do not expect a relevant influence on binding potentials in this region (Matsumoto et al. 1996). Martel et al. concluded that [*carbonyl*-^11^C]WAY-100635 specifically antagonizes the 5-HT_1A_R if adequate doses and concentrations are used. Previous studies have yielded inconsistent results on sex differences in 5-HT_1A_R binding (Parsey et al. 2002; Jovanovic et al. 2008; Stein et al. 2008; Moses-Kolko et al. 2011). Parsey et al. (2002) found significantly higher BP in females compared to males using [*carbonyl*-^11^C]WAY-100635. In a previous publication including the current sample, we did not find any sex differences in 5-HT_1A_R binding (Stein et al. 2008). Not only the differences in the modeling methods but also the age and effects of hormonal status could underlie the diverging results. However, the analysis of sex differences in the correlation of PET and mRNA data is primarily limited by the fact that only one of the six brain donor brains was female. Therefore, there are too few samples to create a comprehensive atlas based exclusively on microarray data from the female brain using our method (Gryglewski et al. 2018).

It must be pointed out that the distribution of mRNA expression throughout the brain might differ from protein density and radioligand-binding sites. The 5-HT_1A_R gene is intronless and the mRNA does not undergo mRNA editing or alternative mRNA splicing (Roth 2006). The gene is highly expressed in the hippocampus, the septum, the raphe nuclei, the neocortex, and the hypothalamus (Hoyer et al. 1994). On a cellular level, apart from serotonergic neurons, the 5-HT_1A_R is also expressed in pyramidal neurons assumed to be glutamatergic in prefrontal and limbic regions (Palchaudhuri and Flugge 2005) and in a GABAergic interneuron subpopulation in the prefrontal cortex (de Almeida and Mengod 2008). On both serotonergic and nonserotonergic neurons, the 5-HT_1A_R is localized predominantly somatodendritically. In line with our results, studies in rats, monkeys, and the human brain have demonstrated a comparable distribution of the 5-HT_1A_R mRNA and radiolabelled binding sites as well as the receptor protein (Roth 2006). The genes found to correlate highly with the 5-HT_1A_R on the cortical surface also showed an overrepresentation in the GO analysis for the somatodendritic compartment (including the 5-HT_1A_R, the 5-HT_2C_R and BDNF), as well as the axon and the plasma membrane bounded cell projection.

**Table 1:** Genes with an SD closest to the BP_ND_ of the 5-HT_1A_R (90th percentile) and a Spearman rho >0.7 on the left cortical surface and in subcortical regions

	Gene name	Entrez ID	RSV (%)	SD	Spearman (BP_ND_ vs. mRNA)	Spearman (mRNA vs. mRNA)
Cortical						
	5-hydroxytryptamine (serotonin) receptor 2C, G protein-coupled	3358	66.51	0.53	0.71	0.86
	5-hydroxytryptamine (serotonin) receptor 1A, G protein-coupled	3350	67.48	0.52	0.72	1.00
	Atonal homolog 7 (Drosophila)	220 202	72.00	0.52	0.70	0.89
	Lymphocyte antigen 6 complex, locus H	4062	77.24	0.49	0.71	0.87
	Natriuretic peptide A	4878	59.83	0.49	0.75	0.82
	Copine VI (neuronal)	9362	90.31	0.45	0.71	0.92
	Annexin A8	653 145	60.95	0.44	0.73	0.84
	ATP/GTP binding protein-like 1	123 624	55.65	0.44	0.76	0.82
	Kinocilin	148 930	41.33	0.43	0.71	0.87
	v-myb myeloblastosis viral oncogene homolog (avian)	4602	50.57	0.42	0.76	0.85
	Complement factor D (adipsin)	1675	67.41	0.42	0.71	0.77
	Serine-rich and transmembrane domain containing 1	400 120	52.13	0.41	0.75	0.80
	Tumor protein p53 inducible protein 11	9537	61.40	0.41	0.76	0.83
	TAF7-like RNA polymerase II, TATA box binding protein (TBP)-associated factor, 50 kDa	54 457	53.73	0.40	0.79	0.88
	Myb/SANT-like DNA-binding domain containing 1	345 222	60.97	0.37	0.72	0.78
	Tumor necrosis factor receptor superfamily, member 11a, NFKB activator	8792	42.48	0.36	0.71	0.72
	Protocadherin 19	57 526	64.88	0.35	0.76	0.85
	Centromere protein W	387 103	75.54	0.35	0.75	0.85
	Sosondowah ankyrin repeat domain family member A	134 548	65.79	0.34	0.74	0.90
	Glycoprotein (transmembrane) nmb	10 457	43.02	0.34	0.72	0.74
Subcortical						
	Neuronal differentiation 2	4761	84.80	2.13	0.81	0.90
	Neuronal differentiation 6	63 974	93.73	2.05	0.71	0.74
	Cholecystokinin	885	56.95	1.73	0.80	0.85
	Complement component 1, q subcomponent-like 3	389 941	61.07	1.67	0.71	0.84
	Brain-derived neurotrophic factor	627	52.91	1.65	0.70	0.83
	Solute carrier family 17 (sodium-dependent inorganic phosphate cotransporter), member 7	57 030	76.39	1.57	0.79	0.86
	LY86 antisense RNA 1 (non-protein coding)	285 780	91.99	1.56	0.75	0.75
	KIAA1239	57 495	63.17	1.50	0.82	0.93
	Transmembrane protein 200A	114 801	50.43	1.37	0.75	0.79
	Calsyntenin 2	64 084	77.86	1.31	0.74	0.91
	Neuronal pentraxin receptor	23 467	78.11	1.29	0.74	0.90
	Tumor necrosis factor, alpha-induced protein 8-like 3	388 121	47.62	1.25	0.75	0.89
	T-box, brain, 1	10 716	91.91	1.21	0.77	0.82
	5-hydroxytryptamine (serotonin) receptor 1A, G protein-coupled	3350	46.63	1.19	0.85	1.00
	Meis homeobox 3	56 917	69.41	1.14	0.75	0.85
	Nephroblastoma overexpressed	4856	69.43	1.11	0.74	0.82
	Harakiri, BCL2 interacting protein (contains only BH3 domain)	8739	84.52	1.10	0.71	0.87
	Transmembrane protein 155	132 332	79.31	1.09	0.70	0.73

Note: RSV, regional structural variability.

**Table 2:** Overrepresented genes on the left cortical hemisphere and in subcortical regions

	GO ID	GO term	Count	*P* value	Genes
Cortical					
Cellular component					
	GO:0030424	Axon	17	<0.01	EXOC6, CPLX3, GPM6A, CORO1A, SNCA, TRPV2, NPTX2, CD200, STX1A, BDNF, NECAB2, PTPRO, GPRIN1, CPNE6, U47924, AP3B2, EPHA5
	GO:0120038	Plasma membrane bounded cell projection part	30	0.04	KNCN, EXOC6, HTR2C, RILPL2, CPLX3, GPM6A, SNCA, PRPH2, U47924, TRPV2, GRM1, NPTX2, AC092324, MPP2, SYTL1, CYP46A1, JPH4, RSPH9, B9D1, CYS1, BDNF, NECAB2, C2orf39, DCDC2, HTR1A, PTPRO, GPRIN1, CPNE6, AP3B2, EPHA5
	GO:0036477	Somatodendritic compartment	21	0.03	KNCN, HTR2C, GPM6A, SNCA, U47924, PYCARD, GRM1, NPTX2, MPP2, CD200, CYP46A1, JPH4, CYGB, BDNF, NECAB2, U47924, HTR1A,PTPRO, CPNE6, EPHA5, RTN4RL1
Subcortical					
Biological process					
	GO:0048666	Neuron development	23	0.02	NEUROD2, NTNG2, CCK, RAPH1, POSTN, WDR5, MAP6, C1QL1, PAX2, ST7, HCN1, TBC1D24, VSTM2L, NPTXR, SYN1, EMX1, BDNF, MAP2, NOV, GPRIN1, NCAM2, RTN4RL2, HPRT1
*Cellular component*					
	GO:0030424	Axon	20	<0.01	NTNG2, CCK, PRKAA2, NOV, MAP6, C1QL1, HCN1, TBC1D24, AP3M2, KIF21B, VSTM2L, NPTXR, DYNLL1, SYN1, BDNF, MAP2, GPRIN1, NCAM2, SLC17A7, RTN4RL2
	GO:0098793	Presynapse	16	0.02	NTNG2, CCK, CADPS2, PFN2, CDH10, C1QL1, CADM3, TBC1D24, SYN1, BDNF, DENND1A, PPFIA2, SLC17A7, DNAJC5, STXBP5, LIN7C
	GO:0031410	Cytoplasmic vesicle	42	0.01	AC011479, RRAGB, SLC39A2, CADPS2, HYOU1, USP10, UBQLN2, RAB2A, ASTN1, MAP6, ANXA8, SLCO4C1, RAB12, CLIP3, CHGB, TBC1D24, AP3M2, TOLLIP, RFTN1, KIF21B, MALL, RNF11, PRSS3, DYNLL1, TECPR1, PDCD6, PRSS2, LYZL4, SYN1, SPRED2, BDNF, EGF, DENND1A, PGRMC1, KLK7, CNIH3, ATP6V1D, SLC17A7, DNAJC5, STXBP5, JAK3, DBH

All *P*-values FDR corrected with *P* < 0.05.

Among the 10 genes correlating highly with the BP_ND_ of the 5-HT_1A_R in both cortical and subcortical regions were *Anxa8* and *BDNF*. BDNF has been repeatedly connected to psychiatric disorders such as schizophrenia, bipolar disorder, depression, and addiction (Autry and Monteggia 2012) and the action of psychopharmacologic drugs. Long-term antidepressant therapies such as SSRIs, tricyclic antidepressants, and electroconvulsive therapy seem to induce increased BDNF mRNA (Autry and Monteggia 2012) and protein expression, and infusion of BDNF into the rat hippocampus has been shown to have antidepressant-like effects (Siuciak et al. 1997). Also, infusions of ketamine, a noncompetitive NMDA-receptor antagonist recently approved as a rapid acting antidepressant, seem to increase BDNF protein levels in the rat hippocampus (Garcia et al. 2008). The BDNF gene codes for a neurotrophin called brain-derived neurotrophic factor, which exists as prepro-BDNF and can be cleaved into pro-BDNF and mature BDNF (Lessmann et al. 2003). Pro-BDNF and mature BDNF seem to have different effects on intracellular signaling pathways (Matsumoto et al. 2008). The mature BDNF unfolds its action through its high-affinity receptor tropomyosin-related kinase B (TrkB) (Autry and Monteggia 2012). Upon binding, intracellular signaling cascades are activated, including the phospholipase-Cγ pathway, the phosphatidyl-inositol 3-kinase pathway, and the MAPK/ERK pathway. Through these pathways, BDNF plays an important role in axonal growth, synaptic plasticity (Yoshii and Constantine-Paton 2010), and learning and memory (Lu et al. 2008). It is further involved in the proper functioning of GABAergic, glutamatergic, dopaminergic, and serotonergic neurons (Pillai 2008). The neurotrophin also appears to influence the morphological differentiation of serotonergic neurons (Martinowich and Lu 2008). Galter and Unsicker (2000) have shown that serotonin upregulates BDNF expression through 5-HT_1A_R-activation and thereby induces promotion and maintenance of differentiated serotonergic neurons in the rat raphe nucleus. It has been proposed that this promotion of the 5HT-phenotype is mediated through a 5-HT_1A_R-induced downregulation of the cAMP-dependent protein kinase A and the activation of the BDNF/TrkB pathway. In BDNF knockout mice, Hensler et al. (2007) demonstrated a reduced 5-HT_1A_R function in the dorsal hippocampus compared to control mice. On the other hand, Trajkovska et al. (2009) did not observe changes in 5-HT_1A_R levels in mouse hippocampal neurons upon short- or long-term exposure with BDNF. Also Vaidya et al. (1997) found no effect of 5-HT_1A_R agonism on BDNF mRNA levels in the hippocampus and neocortex. One explanation might be the agonist used, which also activates 5-HT_7_R that has opposing effects to the 5-HT_1A_R. In general, however, the topological correlation of the BDNF and 5-HT_1A_R mRNA expression levels observed in this analysis may reflect the known interaction of BDNF with the serotonergic, and especially the 5-HT_1A_R, system (Popova et al. 2017) and could be explained through second messenger cascades such as TrkB and downstream signaling.

The *AnxA8*-gene codes for Annexin A8 that forms part of a family of Ca^2+^-regulated phospholipid-binding proteins (Goebeler et al. 2008) that is involved in intracellular processes including cell differentiation, proliferation, and receptor signaling and trafficking (Grewal and Enrich 2009; Monastyrskaya et al. 2009). AnxA8 has been connected to plastic changes of premotor neurons in macaque monkeys following a unilateral lesion of the lateral corticospinal tract (Higo et al. 2018) and has therefore been named a plasticity-related gene. Up until now, to the best of our knowledge, no study has focused on the topological distribution or neuronal gene expression of *AnxA8* in the human brain or its topological coexpression with the 5-HT_1A_R. Previous studies reported on an *AnxA8* expression restricted to certain tissue types, including the skin, lung, liver, and kidney (Pepinsky and Hauptmann 1992; Reutelingsperger et al. 1994). Few studies exist regarding AnxA8 and its role in neuropsychiatric research. Kashem et al. (2009) have shown increased AnxA8 protein expression in risperidone-treated neural stem cells in comparison with haloperidol. Furthermore, Park et al. (2006) have implicated AnxA8 as an activity-regulated gene during long-term potentiation in mice. As a protein that affects the cytoskeleton, AnxA8 could interact with the 5-HT_1A_R in the process of dendritic maturation (Yan et al. 1997) or be involved in the regulation of internalization and intracellular movement of the 5-HT_1A_R through Ca^2+^-mediated mechanisms, as is the case for the epidermal growth factor receptor (Goebeler et al. 2008).

Regarding subcortical regions, the GO analysis revealed overrepresentation of 23 genes involved in the biological process of neuronal development (Table 2), including *NeuroD2*. Apart from a high correlation with the BP_ND_ of the 5-HT_1A_R (ρ = 0.81), *NeuroD2* also showed a similar SD (SD = 2.13) as the BP_ND_ of the 5-HT_1A_R. Alterations in *NeuroD2* have been linked to schizophrenia, where patients with *NeuroD2* polymorphisms showed reduced cognitive functions (Spellmann et al. 2017). The neuronal differentiation family (*NeuroD1*, *NeuroD2*, *NeuroD4*, *NeuroD6*) represents genes that code for neurogenic basic-helix-loop-helix transcription factors. These factors contribute to premature cell cycle arrest and lead to a mature neuronal phenotype by inducing specific gene transcription. Throughout adulthood, these NeuroD transcription factors remain expressed in postmitotic neurons (Olson et al. 2001). In recent years, they have been connected to the survival of a subset of midbrain dopaminergic neurons in the ventral midbrain (Khan et al. 2017) and to amacrine cell subtype identity in the retina (Cherry et al. 2011; Kay et al. 2011). Further, they have been implicated in long-range axogenesis of callosal connections (Bormuth et al. 2013) and a decrease of neuronal excitability in cortical pyramidal neurons (Chen et al. 2016). Moreover, NeuroD2 has been suggested to regulate trophic factors such as BDNF by showing decreased expression of *BDNF* in NeuroD2-null mice cerebella (Olson et al. 2001). This decrease in expression could potentially also affect 5-HT_1A_R-mRNA expression. Various factors influencing the regulation of 5-HT_1A_R-mRNA expression have been defined including REST, Freud-1, Freud-2, as well as allele-specific factors, such as Deaf1 (Albert et al. 2011). A direct involvement of the NeuroD gene family in the expression of the 5-HT_1A_R has not been shown up until now. In support of our findings, *NeuroD2* is also expressed in cortical and hippocampal pyramidal neurons and granule cells in the dentate gyrus (Schwab et al. 1998) and remains expressed in these fully differentiated neuronal subtypes in the adult brain. The proposed coexpression of *NeuroD2* with the 5-HT_1A_R might also reflect the shared inhibitory effects on pyramidal firing. Also, *NeuroD2* could contribute to the 5-HT_1A_R functions of increased neurogenesis and synaptogenesis (Zhang et al. 2016).

## Implications and Future Directions

A dysregulation in processes connected to neuronal development is thought to be one of the underlying causes of psychiatric disorders (Lesch and Waider 2012). In general, the involvement of abovementioned genes reflects the complexity of 5-HTR1A functioning. Using the AHBA, the mRNA expression of the 5-HT_1A_R has been compared to the BP_ND_ of the 5-HT_1A_R (Komorowski et al. 2017) and to other GPCRs (Janusonis 2014). Even though we could not validate the results of, for example, Janusonis (2014) or Sokolina et al. (2017), the current analysis represents one further attempt in the rapidly growing field of imaging transcriptomics (Arnatkevic̆iūtė et al. 2019) to define new functional groups of 5-HT_1A_R signaling. Rather than simple correlations of gene expression that highlight potential functional interactions, future studies should consider the interactions of multiple similarly expressed genes to better reflect the structural composition of a given brain region. For that purpose, unbiased hierarchical clustering approaches have been proposed (Janusonis 2017). Future coexpression analyses could also include other targets for which radiotracers are available, including the MAO-A, serotonin transporter, and serotonin-2A receptor (Komorowski et al. 2017) as well as autoradiography data.

## Limitations

As the analysis is based on the AHBA and PET imaging, the results are influenced by the underlying data and the respective data processing choices (Arnatkevic̆iūtė et al. 2019). Regarding the tracer used, the selectivity of [*carbonyl*-^11^C]WAY-100635 has been shown (Martel et al. 2007). However, the use of the drug-binding site for the correlation could have confounded our results, since it might differ from protein density and be influenced by conformational states. Regarding the AHBA data, it comprises a relatively small sample of six specimens with a great diversity of age, sex, and postmortem intervals that could have impacted the transcriptomic profile obtained. Data from the BrainSpan atlas, for example, show differential expression of two exons of the *NeuroD6* gene across the life span (Mahfouz et al. 2017). The samples analyzed comprise human postmortem microarray probes of a specific gene. It has been suggested that the variability of protein levels can only be explained up to 40% by mRNA levels, with a great impact of translation and post-translational modifications (Janusonis 2017). BDNF, for example, represents the mature protein of the *BDNF* gene. However, the transcript can undergo alternative splicing and several precursors, such as prepro-BDNF and pro-BDNF exist. These subtleties are not taken into account by the AHBA data and the current analysis and would require further work, especially since pro-BDNF and BDNF seem to have opposing effects on neurite growth (Popova et al. 2017). Moreover, investigations of the exon-level transcriptome have revealed spatiotemporal differences in gene expression, especially before birth (Kang et al. 2011). Sun et al. (2005), for example, have shown asymmetric expression of *NeuroD6* in embryonic human brains. These differences seem to normalize with age (Kang et al. 2011). The genetic mosaicism, a concept that has gained attention in recent years (Insel 2014; McConnell et al. 2017; Nishioka et al. 2019), could, however, potentially affect the generalizability of our results. Furthermore, the 5-HT_1A_R can be present not only on excitatory principal neurons but also on inhibitory interneurons (Lesch and Waider 2012) and has been shown to be present in cultured astrocytes (Hirst et al. 1998), a specification that our analysis did not take into account, that could, however, be relevant for differences in gene expression. To improve comparability and sample quality, multiple samples from various cortical and subcortical areas at identical sites and from both hemispheres across subjects would be necessary. Recent technological advances such as next generation sequencing and single-cell whole genome amplification could help in this regard.

## Conclusion

In conclusion, we have correlated predicted whole-brain transcriptomic data from the AHBA with PET data of the 5-HT_1A_R binding to define genes potentially coexpressed with this receptor. The strong correlation between the predicted mRNA expression and the BP_ND_ of the 5-HT_1A_R in cortical and subcortical regions supports the employed methodology. Using our in silico approach, we consolidated the association of “BDNF” and the 5-HT_1A_R and further implicated new genes in serotonergic functioning. Correlating the predicted whole-brain mRNA expression of almost 19 000 genes with the BP_ND_ of the 5-HT_1A_R can be seen as a gene prioritization approach to generate new hypotheses and scientific questions. Given the available data and using computational models, we have reduced the number of genes potentially coexpressed with the 5-HT_1A_R to guide future endeavors to analyze protein interactions, better describe disease pathomechanisms, and lead to new drug targets.

## Funding

Austrian Science Fund (FWF P 27141 to R.L.); Austrian National Bank (OENB P11468 to R.L); Hochschuljubilaeumsstiftung of the City of Vienna (to R.S.); Austrian Science Fund (FWF, DOC 33-B27 to M.M.). The funding sources had no further role in the study design, the collection, analysis and interpretation of data, in the writing of the report nor in the decision to submit the paper for publication.

## Notes

We thank the staff and the diploma students of the Neuroimaging Lab (NIL) at the Department of Psychiatry and Psychotherapy for clinical, scientific, technical, and administrative support. Also, we would like to thank the staff at the Department of Biomedical Imaging and Image-guided Therapy, Division of Nuclear Medicine, for technical support.

## Author Contribution

Without any relevance to this work, S.K. received grants/research support, consulting fees, and/or honoraria within the last 3 years from Angelini, AOP Orphan Pharmaceuticals AG, Celegne GmbH, Eli Lilly, Janssen-Cilag Pharma GmbH, KRKA-Pharma, Lundbeck A/S, Mundipharma, Neuraxpharm, Pfizer, Sanofi, Schwabe, Servier, Shire, Sumitomo Dainippon Pharma Co. Ltd and Takeda. R.L. received conference speaker honorarium within the last 3 years from Shire and research support from Siemens Healthcare regarding clinical research using PET/MR. He is a shareholder of BM Health GmbH since 2019. Without relevance to this work, W.W. received research grants within the last 3 years from Scintomics, Ipsen Pharma, EZAG, and ITG and is a part-time employee of CBmed Ltd, a PPP research center of excellence. All other authors have no biomedical financial interests to disclose and report no potential conflicts of interest. Preliminary findings of this study were submitted to the CINP International Meeting in Athens, Greece, taking place from 3rd to 5th October 2019.

## Supplementary Material

Supplement_bhz341
