# Supplementary material for: Topologically Guided Prioritization of Candidate Gene Transcripts Coexpressed with the 5-HT1A Receptor by Combining In Vivo PET and Allen Human Brain Atlas Data
Source: Cereb Cortex. 2020 Jan 16;30(6):3771–80. doi: 10.1093/cercor/bhz341 (PMC7232988; doi:10.1093/cercor/bhz341)
Supplement: Supplement_bhz341 [file supplement_bhz341.docx]

# Supplement

Table 1 Genes with the highest correlation (r > 0.7) of predicted mRNA expression with the BP_ND_ of the 5-HT_1A_R on the left cortical surface. Spearman correlation coefficients (r) between predicted mRNA expression of 18,686 genes and the BP_ND_ of the 5-HT_1A_R were calculated. RSV=regional structured variability, SD=standard deviation.

| **Gene name** | **Entrez ID** | **RSV (%)** | **SD** | **Spearman (BP_ND_ vs. mRNA)** | **Spearman (mRNA vs. mRNA)** |
| --- | --- | --- | --- | --- | --- |
| exonuclease 3'-5' domain containing 1 | 161829 | 70.28 | 0.70 | 0.71 | 0.86 |
| uncharacterized protein CXorf49-like | 100129291 | 69.14 | 0.69 | 0.73 | 0.90 |
| potassium channel tetramerisation domain containing 4 | 386618 | 81.15 | 0.68 | 0.72 | 0.93 |
| exosome component 6 pseudogene | 392145 | 72.47 | 0.66 | 0.70 | 0.90 |
| achaete-scute complex homolog 2 (Drosophila) | 430 | 71.92 | 0.66 | 0.72 | 0.81 |
| PYD (pyrin domain) containing 1 | 260434 | 70.22 | 0.66 | 0.78 | 0.88 |
| N-terminal EF-hand calcium binding protein 2 | 54550 | 79.44 | 0.64 | 0.73 | 0.93 |
| chromosome 1 open reading frame 187 | 374946 | 67.18 | 0.62 | 0.72 | 0.91 |
| phenylethanolamine N-methyltransferase | 5409 | 85.57 | 0.60 | 0.77 | 0.90 |
| interleukin 13 receptor, alpha 2 | 3598 | 77.59 | 0.58 | 0.73 | 0.88 |
| kallikrein-related peptidase 8 | 11202 | 51.79 | 0.58 | 0.74 | 0.63 |
| radial spoke head 9 homolog (Chlamydomonas) | 221421 | 79.22 | 0.56 | 0.75 | 0.90 |
| 5-hydroxytryptamine (serotonin) receptor 2C, G protein-coupled | 3358 | 66.51 | 0.53 | 0.71 | 0.86 |
| 5-hydroxytryptamine (serotonin) receptor 1A, G protein-coupled | 3350 | 67.48 | 0.52 | 0.72 | 1.00 |
| atonal homolog 7 (Drosophila) | 220202 | 72.00 | 0.52 | 0.70 | 0.89 |
| lymphocyte antigen 6 complex, locus H | 4062 | 77.24 | 0.49 | 0.71 | 0.87 |
| natriuretic peptide A | 4878 | 59.83 | 0.49 | 0.75 | 0.82 |
| copine VI (neuronal) | 9362 | 90.31 | 0.45 | 0.71 | 0.92 |
| annexin A8 | 653145 | 60.95 | 0.44 | 0.73 | 0.84 |
| ATP/GTP binding protein-like 1 | 123624 | 55.65 | 0.44 | 0.76 | 0.82 |
| kinocilin | 148930 | 41.33 | 0.43 | 0.71 | 0.87 |
| v-myb myeloblastosis viral oncogene homolog (avian) | 4602 | 50.57 | 0.42 | 0.76 | 0.85 |
| complement factor D (adipsin) | 1675 | 67.41 | 0.42 | 0.71 | 0.77 |
| serine-rich and transmembrane domain containing 1 | 400120 | 52.13 | 0.41 | 0.75 | 0.80 |
| tumor protein p53 inducible protein 11 | 9537 | 61.40 | 0.41 | 0.76 | 0.83 |
| TAF7-like RNA polymerase II, TATA box binding protein (TBP)-associated factor, 50kDa | 54457 | 53.73 | 0.40 | 0.79 | 0.88 |
| Myb/SANT-like DNA-binding domain containing 1 | 345222 | 60.97 | 0.37 | 0.72 | 0.78 |
| tumor necrosis factor receptor superfamily, member 11a, NFKB activator | 8792 | 42.48 | 0.36 | 0.71 | 0.72 |
| protocadherin 19 | 57526 | 64.88 | 0.35 | 0.76 | 0.85 |
| centromere protein W | 387103 | 75.54 | 0.35 | 0.75 | 0.85 |
| sosondowah ankyrin repeat domain family member A | 134548 | 65.79 | 0.34 | 0.74 | 0.90 |
| glycoprotein (transmembrane) nmb | 10457 | 43.02 | 0.34 | 0.72 | 0.74 |
| neuronal pentraxin II | 4885 | 33.19 | 0.34 | 0.71 | 0.69 |
| zinc finger, CCHC domain containing 18 | 644353 | 43.32 | 0.34 | 0.76 | 0.87 |
| chromosome 4 open reading frame 45 | 152940 | 47.60 | 0.34 | 0.79 | 0.86 |
| complexin 3 | 594855 | 52.62 | 0.33 | 0.71 | 0.77 |
| glycine receptor, alpha 2 | 2742 | 29.17 | 0.33 | 0.74 | 0.83 |
| RAS protein activator like 1 (GAP1 like) | 8437 | 51.15 | 0.33 | 0.71 | 0.89 |
| basic leucine zipper transcription factor, ATF-like 3 | 55509 | 58.27 | 0.32 | 0.73 | 0.83 |
| protein kinase (cAMP-dependent, catalytic) inhibitor alpha | 5569 | 72.19 | 0.31 | 0.71 | 0.92 |
| nuclear protein, transcriptional regulator, 1-like | 389493 | 50.54 | 0.31 | 0.74 | 0.88 |
| uncharacterized LOC284861 | 284861 | 20.06 | 0.30 | 0.78 | 0.72 |
| pregnancy up-regulated non-ubiquitously expressed CaM kinase | 139728 | 49.92 | 0.30 | 0.71 | 0.84 |
| synaptotagmin-like 1 | 84958 | 50.62 | 0.30 | 0.71 | 0.82 |
| KIAA1239 | 57495 | 54.99 | 0.30 | 0.75 | 0.86 |
| parathyroid hormone-like hormone | 5744 | 52.77 | 0.30 | 0.70 | 0.80 |
| troponin I type 3 (cardiac) | 7137 | 37.76 | 0.29 | 0.75 | 0.77 |
| chromosome 16 open reading frame 73 | 254528 | 18.83 | 0.29 | 0.71 | 0.79 |
| thiosulfate sulfurtransferase (rhodanese)-like domain containing 1 | 100131187 | 57.59 | 0.29 | 0.72 | 0.78 |
| hippocalcin like 4 | 51440 | 69.13 | 0.29 | 0.74 | 0.90 |
| synuclein, alpha (non A4 component of amyloid precursor) | 6622 | 60.07 | 0.29 | 0.71 | 0.81 |
| dapper, antagonist of beta-catenin, homolog 1 (Xenopus laevis) | 51339 | 47.76 | 0.28 | 0.77 | 0.88 |
| FLJ45248 protein | 401472 | 32.56 | 0.28 | 0.72 | 0.78 |
| doublecortin domain containing 2 | 51473 | 41.24 | 0.28 | 0.77 | 0.77 |
| calcium channel, voltage-dependent, L type, alpha 1S subunit | 779 | 33.04 | 0.28 | 0.75 | 0.86 |
| MYCN opposite strand/antisense RNA (non-protein coding) | 10408 | 34.13 | 0.28 | 0.73 | 0.81 |
| TBC1 domain family, member 26 | 353149 | 61.98 | 0.28 | 0.73 | 0.87 |
| interleukin 12 receptor, beta 2 | 3595 | 31.94 | 0.28 | 0.75 | 0.84 |
| profilin 3 | 345456 | 23.07 | 0.28 | 0.78 | 0.78 |
| sorting nexin 7 | 51375 | 75.04 | 0.28 | 0.72 | 0.84 |
| HRAS-like suppressor family, member 5 | 117245 | 32.60 | 0.28 | 0.78 | 0.83 |
| immunoglobulin superfamily, member 22 | 283284 | 47.50 | 0.27 | 0.78 | 0.89 |
| phosphoglucomutase 2-like 1 | 283209 | 52.03 | 0.27 | 0.72 | 0.78 |
| protein kinase (cAMP-dependent, catalytic) inhibitor beta | 5570 | 50.37 | 0.27 | 0.77 | 0.72 |
| taxilin beta | 167838 | 31.44 | 0.27 | 0.78 | 0.85 |
| histone linker H1 domain, spermatid-specific 1, pseudogene | 373861 | 50.88 | 0.26 | 0.78 | 0.86 |
| coronin, actin binding protein, 1A | 11151 | 35.61 | 0.26 | 0.71 | 0.75 |
| complement component 1, r subcomponent | 715 | 54.76 | 0.26 | 0.74 | 0.86 |
| TBC1 domain family, member 28 | 254272 | 17.95 | 0.26 | 0.74 | 0.86 |
| PYD and CARD domain containing | 29108 | 30.02 | 0.26 | 0.76 | 0.74 |
| B9 protein domain 1 | 27077 | 72.04 | 0.26 | 0.74 | 0.85 |
| dihydrodiol dehydrogenase (dimeric) | 27294 | 50.34 | 0.25 | 0.77 | 0.88 |
| protein tyrosine phosphatase, receptor type, R | 5801 | 58.23 | 0.25 | 0.75 | 0.84 |
| cytoglobin | 114757 | 19.79 | 0.25 | 0.75 | 0.70 |
| Rab interacting lysosomal protein-like 2 | 196383 | 73.17 | 0.25 | 0.71 | 0.87 |
| protein tyrosine phosphatase, non-receptor type 3 | 5774 | 52.64 | 0.25 | 0.71 | 0.62 |
| RAS-like, family 11, member B | 65997 | 48.07 | 0.25 | 0.71 | 0.77 |
| serine/threonine/tyrosine kinase 1 | 55359 | 48.39 | 0.25 | 0.76 | 0.83 |
| pyrimidinergic receptor P2Y, G-protein coupled, 6 | 5031 | 27.29 | 0.25 | 0.76 | 0.68 |
| family with sequence similarity 19 (chemokine (C-C motif)-like), member A1 | 407738 | 42.35 | 0.24 | 0.76 | 0.88 |
| calcium/calmodulin-dependent protein kinase II delta | 817 | 71.55 | 0.24 | 0.77 | 0.88 |
| family with sequence similarity 26, member F | 441168 | 49.17 | 0.24 | 0.74 | 0.84 |
| nucleolar protein 4 | 8715 | 50.33 | 0.24 | 0.75 | 0.85 |
| OTU domain, ubiquitin aldehyde binding 2 | 78990 | 32.24 | 0.24 | 0.75 | 0.84 |
| chemokine (C-C motif) ligand 28 | 56477 | 18.99 | 0.24 | 0.74 | 0.75 |
| erythrocyte membrane protein band 4.1 like 4B | 54566 | 38.59 | 0.24 | 0.72 | 0.81 |
| brain-derived neurotrophic factor | 627 | 24.51 | 0.23 | 0.78 | 0.83 |
| chromosome 19 open reading frame 76 | 199800 | 44.33 | 0.23 | 0.73 | 0.86 |
| histone cluster 1, H2bk | 85236 | 53.56 | 0.23 | 0.72 | 0.80 |
| olfactory receptor, family 6, subfamily C, member 2 | 341416 | 15.32 | 0.23 | 0.71 | 0.79 |
| delta-like 3 (Drosophila) | 10683 | 26.43 | 0.23 | 0.70 | 0.76 |
| numb homolog (Drosophila)-like | 9253 | 12.45 | 0.22 | 0.80 | 0.84 |
| phosphodiesterase 2A, cGMP-stimulated | 5138 | 37.73 | 0.22 | 0.71 | 0.83 |
| FYVE, RhoGEF and PH domain containing 3 | 89846 | 38.42 | 0.22 | 0.73 | 0.73 |
| immunoglobulin superfamily, member 10 | 285313 | 18.81 | 0.22 | 0.74 | 0.80 |
| ribosomal protein L39-like | 116832 | 46.29 | 0.22 | 0.79 | 0.81 |
| EH domain binding protein 1-like 1 | 254102 | 39.33 | 0.21 | 0.77 | 0.79 |
| synaptotagmin-like 5 | 94122 | 20.86 | 0.21 | 0.74 | 0.88 |
| cytochrome P450, family 46, subfamily A, polypeptide 1 | 10858 | 36.52 | 0.21 | 0.72 | 0.82 |
| peripherin 2 (retinal degeneration, slow) | 5961 | 63.94 | 0.21 | 0.78 | 0.82 |
| ISG15 ubiquitin-like modifier | 9636 | 51.66 | 0.21 | 0.74 | 0.82 |
| CaM kinase-like vesicle-associated | 79012 | 47.00 | 0.21 | 0.76 | 0.87 |
| protein tyrosine phosphatase, receptor type, O | 5800 | 34.81 | 0.21 | 0.74 | 0.80 |
| glutamate receptor, metabotropic 1 | 2911 | 38.63 | 0.21 | 0.75 | 0.81 |
| RMI2, RecQ mediated genome instability 2, homolog (S. cerevisiae) | 116028 | 39.30 | 0.21 | 0.79 | 0.85 |
| EPH receptor A5 | 2044 | 34.05 | 0.20 | 0.73 | 0.72 |
| reticulon 4 receptor-like 1 | 146760 | 32.08 | 0.20 | 0.71 | 0.67 |
| latent transforming growth factor beta binding protein 4 | 8425 | 46.01 | 0.20 | 0.72 | 0.86 |
| alpha-1-B glycoprotein | 1 | 34.57 | 0.20 | 0.74 | 0.85 |
| GSG1-like | 146395 | 27.42 | 0.20 | 0.74 | 0.73 |
| UDP-N-acetyl-alpha-D-galactosamine:polypeptide N-acetylgalactosaminyltransferase-like 4 | 374378 | 28.85 | 0.20 | 0.77 | 0.81 |
| aspartate beta-hydroxylase domain containing 2 | 57168 | 43.06 | 0.20 | 0.77 | 0.70 |
| G protein regulated inducer of neurite outgrowth 1 | 114787 | 51.54 | 0.20 | 0.75 | 0.90 |
| shisa homolog 9 (Xenopus laevis) | 729993 | 53.58 | 0.19 | 0.70 | 0.89 |
| prostaglandin F receptor (FP) | 5737 | 9.05 | 0.19 | 0.73 | 0.79 |
| sphingomyelin phosphodiesterase 2, neutral membrane (neutral sphingomyelinase) | 6610 | 25.59 | 0.19 | 0.76 | 0.77 |
| DENN/MADD domain containing 1C | 79958 | 21.52 | 0.19 | 0.70 | 0.73 |
| uncharacterized FLJ40288 | 286023 | 51.87 | 0.19 | 0.72 | 0.83 |
| cadherin 4, type 1, R-cadherin (retinal) | 1002 | 45.60 | 0.19 | 0.73 | 0.85 |
| long intergenic non-protein coding RNA 152 | 112597 | 43.34 | 0.19 | 0.72 | 0.73 |
| DDB1 and CUL4 associated factor 15 | 90379 | 27.18 | 0.18 | 0.75 | 0.77 |
| dynein, axonemal, heavy chain 6 | 1768 | 30.03 | 0.18 | 0.74 | 0.85 |
| sorbin and SH3 domain containing 2 | 8470 | 43.60 | 0.18 | 0.70 | 0.88 |
| T-cell activation RhoGTPase activating protein | 117289 | 22.40 | 0.18 | 0.71 | 0.59 |
| solute carrier family 39 (zinc transporter), member 4 | 55630 | 46.92 | 0.18 | 0.78 | 0.84 |
| tumor necrosis factor, alpha-induced protein 8-like 1 | 126282 | 19.97 | 0.18 | 0.70 | 0.70 |
| transient receptor potential cation channel, subfamily V, member 2 | 51393 | 55.55 | 0.17 | 0.71 | 0.71 |
| uridine phosphorylase 2 | 151531 | 32.45 | 0.17 | 0.76 | 0.87 |
| Kruppel-like factor 10 | 7071 | 25.95 | 0.17 | 0.75 | 0.84 |
| myristoylated alanine-rich protein kinase C substrate | 4082 | 19.15 | 0.17 | 0.72 | 0.84 |
| HOP homeobox | 84525 | 32.63 | 0.16 | 0.72 | 0.81 |
| cAMP-regulated phosphoprotein, 19kDa | 10776 | 38.89 | 0.16 | 0.71 | 0.75 |
| solute carrier family 16, member 8 (monocarboxylic acid transporter 3) | 23539 | 50.12 | 0.16 | 0.70 | 0.85 |
| FAD-dependent oxidoreductase domain containing 2 | 80020 | 45.88 | 0.16 | 0.73 | 0.85 |
| beaded filament structural protein 1, filensin | 631 | 44.05 | 0.16 | 0.80 | 0.82 |
| MKL/myocardin-like 2 | 57496 | 43.02 | 0.16 | 0.72 | 0.87 |
| NHL repeat containing 1 | 378884 | 44.63 | 0.15 | 0.73 | 0.85 |
| coiled-coil domain containing 164 | 92749 | 38.90 | 0.15 | 0.72 | 0.76 |
| microtubule associated serine/threonine kinase 3 | 23031 | 24.39 | 0.15 | 0.73 | 0.78 |
| syntaxin 1A (brain) | 6804 | 38.39 | 0.15 | 0.70 | 0.82 |
| uncharacterized LOC440101 | 440101 | 34.42 | 0.14 | 0.74 | 0.81 |
| solute carrier family 29 (nucleoside transporters), member 3 | 55315 | 28.21 | 0.14 | 0.77 | 0.83 |
| small nuclear RNA activating complex, polypeptide 5, 19kDa | 10302 | 36.40 | 0.14 | 0.72 | 0.70 |
| ATP-binding cassette, sub-family A (ABC1), member 17, pseudogene | 650655 | 37.58 | 0.14 | 0.74 | 0.84 |
| cystin 1 | 192668 | 38.30 | 0.14 | 0.76 | 0.84 |
| synaptotagmin-like 2 | 54843 | 32.83 | 0.14 | 0.72 | 0.78 |
| neurexin 2 | 9379 | 17.54 | 0.14 | 0.76 | 0.74 |
| peptidylprolyl isomerase (cyclophilin)-like 6 | 285755 | 39.17 | 0.14 | 0.71 | 0.82 |
| tripartite motif containing 27 | 5987 | 47.42 | 0.14 | 0.71 | 0.81 |
| zinc finger protein 215 | 7762 | 22.34 | 0.14 | 0.74 | 0.72 |
| glycoprotein M6A | 2823 | 37.69 | 0.14 | 0.77 | 0.89 |
| CD200 molecule | 4345 | 23.41 | 0.14 | 0.76 | 0.81 |
| WD repeat domain 54 | 84058 | 43.51 | 0.14 | 0.71 | 0.75 |
| zinc finger protein 436 | 80818 | 43.17 | 0.14 | 0.75 | 0.89 |
| transmembrane protein 132A | 54972 | 30.56 | 0.13 | 0.73 | 0.88 |
| uncharacterized LOC100287628 | 100287628 | 37.82 | 0.13 | 0.75 | 0.85 |
| nucleophosmin/nucleoplasmin 3 | 10360 | 14.39 | 0.13 | 0.77 | 0.76 |
| spindle and centriole associated protein 1 | 152185 | 23.57 | 0.13 | 0.71 | 0.79 |
| uncharacterized LOC644662 | 644662 | 35.74 | 0.13 | 0.71 | 0.69 |
| IKBKB interacting protein | 121457 | 28.78 | 0.13 | 0.75 | 0.86 |
| MAD2 mitotic arrest deficient-like 2 (yeast) | 10459 | 30.21 | 0.13 | 0.70 | 0.81 |
| tumor suppressor candidate 3 | 7991 | 42.72 | 0.13 | 0.71 | 0.84 |
| diacylglycerol kinase, alpha 80kDa | 1606 | 24.53 | 0.13 | 0.77 | 0.74 |
| SRR1 domain containing | 402055 | 47.45 | 0.13 | 0.76 | 0.78 |
| serine/arginine-rich splicing factor 12 | 135295 | 25.69 | 0.13 | 0.74 | 0.79 |
| collapsin response mediator protein 1 | 1400 | 29.89 | 0.13 | 0.72 | 0.85 |
| exocyst complex component 6 | 54536 | 48.04 | 0.12 | 0.74 | 0.91 |
| membrane protein, palmitoylated 2 (MAGUK p55 subfamily member 2) | 4355 | 24.62 | 0.12 | 0.73 | 0.74 |
| - | 727726 | 18.24 | 0.12 | 0.70 | 0.66 |
| NEL-like 2 (chicken) | 4753 | 32.03 | 0.12 | 0.73 | 0.89 |
| melanoma antigen family H, 1 | 28986 | 28.48 | 0.12 | 0.71 | 0.78 |
| lysophosphatidylcholine acyltransferase 3 | 10162 | 25.20 | 0.12 | 0.71 | 0.68 |
| septin 5 | 5413 | 20.89 | 0.12 | 0.70 | 0.76 |
| 3-hydroxy-3-methylglutaryl-CoA reductase | 3156 | 17.66 | 0.11 | 0.70 | 0.87 |
| Ras-related GTP binding D | 58528 | 27.39 | 0.11 | 0.70 | 0.80 |
| uncharacterized LOC729683 | 729683 | 25.07 | 0.11 | 0.72 | 0.81 |
| uncharacterized LOC147727 | 147727 | 17.20 | 0.11 | 0.71 | 0.84 |
| brain-specific angiogenesis inhibitor 3 | 577 | 30.90 | 0.11 | 0.71 | 0.83 |
| cellular repressor of E1A-stimulated genes 1 | 8804 | 35.45 | 0.10 | 0.70 | 0.78 |
| X-linked Kx blood group (McLeod syndrome) | 7504 | 21.50 | 0.10 | 0.73 | 0.75 |
| leucine carboxyl methyltransferase 1 pseudogene | 554206 | 21.42 | 0.10 | 0.71 | 0.71 |
| chromatin assembly factor 1, subunit B (p60) | 8208 | 23.93 | 0.10 | 0.79 | 0.74 |
| ring finger protein 2 | 6045 | 30.67 | 0.10 | 0.77 | 0.80 |
| tripartite motif containing 24 | 8805 | 33.70 | 0.10 | 0.73 | 0.80 |
| adaptor-related protein complex 3, beta 2 subunit | 8120 | 28.33 | 0.10 | 0.71 | 0.77 |
| junctophilin 4 | 84502 | 17.28 | 0.09 | 0.76 | 0.75 |
| nicolin 1 | 84276 | 38.56 | 0.09 | 0.70 | 0.68 |
| activin A receptor, type IIB | 93 | 15.90 | 0.09 | 0.74 | 0.83 |
| leucine zipper, down-regulated in cancer 1-like | 84247 | 18.49 | 0.09 | 0.71 | 0.73 |
| transmembrane protein 234 | 56063 | 26.84 | 0.09 | 0.70 | 0.77 |
| DDB1 and CUL4 associated factor 4 | 26094 | 18.79 | 0.09 | 0.73 | 0.76 |
| chromosome 5 open reading frame 55 | 116349 | 23.08 | 0.09 | 0.73 | 0.81 |
| vesicular, overexpressed in cancer, prosurvival protein 1 | 81552 | 24.98 | 0.08 | 0.72 | 0.80 |
| mitochondrial ribosomal protein L49 | 740 | 15.11 | 0.08 | 0.72 | 0.80 |
| transmembrane protein 18 | 129787 | 28.01 | 0.08 | 0.71 | 0.79 |
| integrin alpha FG-GAP repeat containing 1 | 81533 | 19.88 | 0.07 | 0.73 | 0.83 |
| PHD finger protein 13 | 148479 | 35.36 | 0.07 | 0.74 | 0.86 |
| zinc finger protein 26 | 7574 | 21.66 | 0.07 | 0.74 | 0.75 |
| WD repeat domain 92 | 116143 | 21.68 | 0.07 | 0.72 | 0.70 |

Table 2 Genes with the highest correlation (r > 0.7) of predicted mRNA expression with the BP_ND_ of the 5-HT_1A_R in subcortical regions. Spearman correlation coefficients (r) between predicted mRNA expression of 18,686 genes and the BP_ND_ of the 5-HT_1A_R was calculated. RSV=regional structured variability, SD=standard deviation.

| **Gene name** | **Entrez ID** | **RSV (%)** | **SD** | **Spearman (BP_ND_ vs. mRNA)** | **Spearman (mRNA vs. mRNA)** |
| --- | --- | --- | --- | --- | --- |
| neuronal differentiation 2 | 4761 | 84.80 | 2.13 | 0.81 | 0.90 |
| neuronal differentiation 6 | 63974 | 93.73 | 2.05 | 0.71 | 0.74 |
| cholecystokinin | 885 | 56.95 | 1.73 | 0.80 | 0.85 |
| complement component 1, q subcomponent-like 3 | 389941 | 61.07 | 1.67 | 0.71 | 0.84 |
| brain-derived neurotrophic factor | 627 | 52.91 | 1.65 | 0.70 | 0.83 |
| solute carrier family 17 (sodium-dependent inorganic phosphate cotransporter), member 7 | 57030 | 76.39 | 1.57 | 0.79 | 0.86 |
| LY86 antisense RNA 1 (non-protein coding) | 285780 | 91.99 | 1.56 | 0.75 | 0.75 |
| KIAA1239 | 57495 | 63.17 | 1.50 | 0.82 | 0.93 |
| transmembrane protein 200A | 114801 | 50.43 | 1.37 | 0.75 | 0.79 |
| calsyntenin 2 | 64084 | 77.86 | 1.31 | 0.74 | 0.91 |
| neuronal pentraxin receptor | 23467 | 78.11 | 1.29 | 0.74 | 0.90 |
| tumor necrosis factor, alpha-induced protein 8-like 3 | 388121 | 47.62 | 1.25 | 0.75 | 0.89 |
| T-box, brain, 1 | 10716 | 91.91 | 1.21 | 0.77 | 0.82 |
| 5-hydroxytryptamine (serotonin) receptor 1A, G protein-coupled | 3350 | 46.63 | 1.19 | 0.85 | 1.00 |
| Meis homeobox 3 | 56917 | 69.41 | 1.14 | 0.75 | 0.85 |
| nephroblastoma overexpressed | 4856 | 69.43 | 1.11 | 0.74 | 0.82 |
| harakiri, BCL2 interacting protein (contains only BH3 domain) | 8739 | 84.52 | 1.10 | 0.71 | 0.87 |
| transmembrane protein 155 | 132332 | 79.31 | 1.09 | 0.70 | 0.73 |
| phosphodiesterase 1A, calmodulin-dependent | 5136 | 60.24 | 1.09 | 0.72 | 0.86 |
| metallophosphoesterase domain containing 1 | 758 | 65.46 | 1.07 | 0.75 | 0.80 |
| cellular repressor of E1A-stimulated genes 2 | 200407 | 67.57 | 1.07 | 0.84 | 0.92 |
| leucine rich repeat containing 2 | 79442 | 52.86 | 1.04 | 0.77 | 0.93 |
| PR domain containing 8 | 56978 | 74.76 | 1.01 | 0.80 | 0.87 |
| tripartite motif containing 54 | 57159 | 55.60 | 1.01 | 0.71 | 0.78 |
| annexin A8 | 653145 | 61.43 | 1.01 | 0.74 | 0.78 |
| chromosome 1 open reading frame 135 | 79000 | 61.64 | 1.01 | 0.76 | 0.87 |
| phosphoglucomutase 2-like 1 | 283209 | 59.87 | 1.00 | 0.71 | 0.84 |
| major facilitator superfamily domain containing 4 | 148808 | 80.32 | 0.99 | 0.74 | 0.85 |
| chromosome 3 open reading frame 80 | 401097 | 68.66 | 0.97 | 0.82 | 0.90 |
| sterile alpha motif domain containing 3 | 154075 | 46.37 | 0.97 | 0.81 | 0.92 |
| melanoma associated antigen (mutated) 1-like 1 | 139221 | 30.17 | 0.95 | 0.77 | 0.91 |
| Ca++-dependent secretion activator 2 | 93664 | 56.39 | 0.94 | 0.80 | 0.89 |
| R-spondin 2 | 340419 | 51.44 | 0.93 | 0.75 | 0.86 |
| raftlin, lipid raft linker 1 | 23180 | 65.75 | 0.93 | 0.73 | 0.82 |
| periostin, osteoblast specific factor | 10631 | 55.21 | 0.90 | 0.73 | 0.77 |
| chromosome 4 open reading frame 45 | 152940 | 58.42 | 0.89 | 0.71 | 0.84 |
| chromodomain helicase DNA binding protein 5 | 26038 | 63.05 | 0.88 | 0.72 | 0.88 |
| lysozyme-like 4 | 131375 | 81.83 | 0.86 | 0.76 | 0.73 |
| G protein regulated inducer of neurite outgrowth 1 | 114787 | 49.27 | 0.85 | 0.75 | 0.90 |
| ATP-binding cassette, sub-family C (CFTR/MRP), member 12 | 94160 | 73.48 | 0.84 | 0.73 | 0.74 |
| retinoic acid receptor responder (tazarotene induced) 1 | 5918 | 58.52 | 0.84 | 0.74 | 0.89 |
| chromogranin B (secretogranin 1) | 1114 | 43.52 | 0.81 | 0.71 | 0.84 |
| hyperpolarization activated cyclic nucleotide-gated potassium channel 1 | 348980 | 47.37 | 0.79 | 0.80 | 0.88 |
| tyrosine 3-monooxygenase/tryptophan 5-monooxygenase activation protein, eta polypeptide | 7533 | 51.24 | 0.78 | 0.71 | 0.84 |
| V-set and transmembrane domain containing 2 like | 128434 | 55.30 | 0.76 | 0.74 | 0.84 |
| synapsin I | 6853 | 67.83 | 0.76 | 0.73 | 0.85 |
| protease, serine, 3 pseudogene 2 | 154754 | 46.84 | 0.70 | 0.75 | 0.76 |
| gastrin | 2520 | 50.62 | 0.70 | 0.80 | 0.83 |
| cornichon homolog 3 (Drosophila) | 149111 | 51.63 | 0.70 | 0.72 | 0.85 |
| tetratricopeptide repeat domain 9B | 148014 | 48.84 | 0.70 | 0.79 | 0.86 |
| glycophorin E (MNS blood group) | 2996 | 45.25 | 0.70 | 0.85 | 0.90 |
| zinc and ring finger 4 | 148066 | 69.28 | 0.68 | 0.74 | 0.79 |
| neuronal PAS domain protein 1 | 4861 | 33.49 | 0.68 | 0.70 | 0.79 |
| reticulon 4 receptor-like 2 | 349667 | 68.63 | 0.68 | 0.78 | 0.79 |
| empty spiracles homeobox 1 | 2016 | 62.30 | 0.67 | 0.73 | 0.78 |
| kallikrein-related peptidase 7 | 5650 | 34.55 | 0.66 | 0.80 | 0.77 |
| EF-hand domain family, member D2 | 79180 | 59.07 | 0.66 | 0.74 | 0.87 |
| betaine--homocysteine S-methyltransferase | 635 | 49.52 | 0.64 | 0.83 | 0.87 |
| heat shock 27kDa protein 3 | 8988 | 53.74 | 0.64 | 0.74 | 0.65 |
| paired box 2 | 5076 | 84.34 | 0.63 | 0.78 | 0.87 |
| c-Maf inducing protein | 80790 | 40.96 | 0.63 | 0.77 | 0.93 |
| chromosome 14 open reading frame 162 | 56936 | 39.50 | 0.63 | 0.71 | 0.91 |
| neural cell adhesion molecule 2 | 4685 | 50.45 | 0.62 | 0.72 | 0.79 |
| netrin G2 | 84628 | 39.27 | 0.62 | 0.76 | 0.73 |
| aryl-hydrocarbon receptor repressor | 57491 | 49.87 | 0.60 | 0.77 | -0.54 |
| profilin 2 | 5217 | 58.48 | 0.58 | 0.73 | 0.83 |
| parathyroid hormone 2 receptor | 5746 | 34.08 | 0.58 | 0.71 | 0.84 |
| chromosome 16 open reading frame 93 | 90835 | 43.81 | 0.57 | 0.72 | 0.89 |
| glycophorin B (MNS blood group) | 2994 | 42.76 | 0.57 | 0.80 | 0.89 |
| protease, serine, 3 | 5646 | 34.35 | 0.57 | 0.71 | 0.70 |
| family with sequence similarity 75, member A4 | 642629 | 45.75 | 0.56 | 0.71 | 0.76 |
| protease, serine, 2 (trypsin 2) | 5645 | 53.15 | 0.55 | 0.76 | 0.81 |
| SLC26A4 antisense RNA 1 (non-protein coding) | 286002 | 53.93 | 0.55 | 0.81 | 0.73 |
| N-ethylmaleimide-sensitive factor attachment protein, beta | 63908 | 38.15 | 0.55 | 0.71 | 0.84 |
| ERC2 intronic transcript 1 (non-protein coding) | 711 | 33.75 | 0.53 | 0.80 | 0.87 |
| gap junction protein, beta 5, 31.1kDa | 2709 | 42.53 | 0.52 | 0.77 | 0.89 |
| uncharacterized LOC401232 | 401232 | 25.82 | 0.52 | 0.76 | 0.84 |
| mastermind-like domain containing 1 | 10046 | 20.93 | 0.52 | 0.70 | 0.85 |
| two pore channel 3 pseudogene | 440895 | 32.58 | 0.50 | 0.77 | 0.77 |
| septin 3 | 55964 | 44.93 | 0.50 | 0.73 | 0.82 |
| solute carrier organic anion transporter family, member 2A1 | 6578 | 47.38 | 0.50 | 0.77 | 0.84 |
| transmembrane protein 150C | 441027 | 35.81 | 0.48 | 0.76 | 0.92 |
| tumor necrosis factor, alpha-induced protein 8-like 1 | 126282 | 45.60 | 0.47 | 0.86 | 0.91 |
| thyrotropin-releasing hormone degrading enzyme | 29953 | 63.48 | 0.46 | 0.74 | 0.75 |
| family with sequence similarity 174, member B | 400451 | 55.76 | 0.45 | 0.71 | 0.87 |
| Ras association (RalGDS/AF-6) and pleckstrin homology domains 1 | 65059 | 44.94 | 0.45 | 0.76 | 0.92 |
| chromosome 16 open reading frame 45 | 89927 | 42.53 | 0.45 | 0.78 | 0.86 |
| cadherin 10, type 2 (T2-cadherin) | 1008 | 42.69 | 0.44 | 0.74 | 0.85 |
| syntaxin binding protein 5 (tomosyn) | 134957 | 44.66 | 0.43 | 0.81 | 0.88 |
| single stranded DNA binding protein 3 | 23648 | 49.41 | 0.43 | 0.71 | 0.64 |
| kinesin family member 21B | 23046 | 47.41 | 0.43 | 0.71 | 0.84 |
| dihydroxyacetone kinase 2 homolog (S. cerevisiae) | 26007 | 42.06 | 0.42 | 0.75 | 0.79 |
| ADAM metallopeptidase with thrombospondin type 1 motif, 16 | 170690 | 56.55 | 0.42 | 0.71 | 0.74 |
| mal, T-cell differentiation protein-like | 7851 | 38.90 | 0.41 | 0.73 | 0.75 |
| 3-hydroxy-3-methylglutaryl-CoA reductase | 3156 | 41.36 | 0.41 | 0.78 | 0.83 |
| sarcoglycan, gamma (35kDa dystrophin-associated glycoprotein) | 6445 | 51.89 | 0.41 | 0.76 | 0.80 |
| progesterone receptor membrane component 1 | 10857 | 12.86 | 0.41 | 0.72 | 0.88 |
| brain expressed, X-linked 5 | 340542 | 41.83 | 0.40 | 0.76 | 0.89 |
| TBC1 domain family, member 24 | 57465 | 47.67 | 0.39 | 0.75 | 0.88 |
| nudix (nucleoside diphosphate linked moiety X)-type motif 4 pseudogene 1 | 440672 | 26.64 | 0.39 | 0.74 | 0.49 |
| cytidine monophosphate N-acetylneuraminic acid synthetase | 55907 | 43.45 | 0.39 | 0.72 | 0.86 |
| solute carrier family 39 (zinc transporter), member 2 | 29986 | 52.78 | 0.38 | 0.74 | 0.83 |
| reticulon 1 | 6252 | 27.84 | 0.38 | 0.77 | 0.85 |
| mitogen-activated protein kinase kinase kinase kinase 3 | 8491 | 54.85 | 0.37 | 0.75 | 0.87 |
| solute carrier organic anion transporter family, member 4C1 | 353189 | 21.88 | 0.37 | 0.86 | 0.93 |
| Janus kinase 3 | 3718 | 42.20 | 0.35 | 0.74 | 0.86 |
| spermatogenesis associated, serine-rich 2 | 65244 | 18.27 | 0.35 | 0.73 | 0.77 |
| family with sequence similarity 213, member B | 127281 | 38.70 | 0.35 | 0.70 | 0.87 |
| astrotactin 1 | 460 | 56.55 | 0.35 | 0.71 | 0.85 |
| microtubule-associated protein 2 | 4133 | 24.92 | 0.35 | 0.72 | 0.82 |
| toll interacting protein | 54472 | 37.27 | 0.34 | 0.72 | 0.76 |
| hypoxanthine phosphoribosyltransferase 1 | 3251 | 35.78 | 0.34 | 0.74 | 0.80 |
| SATB homeobox 1 | 6304 | 32.66 | 0.34 | 0.71 | 0.81 |
| tryptophan hydroxylase 2 | 121278 | 38.88 | 0.33 | 0.71 | 0.89 |
| mediator complex subunit 15 | 51586 | 14.84 | 0.32 | 0.74 | 0.80 |
| RAB12, member RAS oncogene family | 201475 | 38.32 | 0.32 | 0.78 | 0.87 |
| protein tyrosine phosphatase, receptor type, f polypeptide (PTPRF), interacting protein (liprin), alpha 2 | 8499 | 76.05 | 0.32 | 0.70 | 0.82 |
| CDC42 small effector 2 | 56990 | 44.62 | 0.32 | 0.72 | 0.80 |
| uncharacterized LOC642852 | 642852 | 28.89 | 0.32 | 0.74 | 0.90 |
| Ras-related GTP binding B | 10325 | 18.94 | 0.31 | 0.71 | 0.89 |
| ATP/GTP binding protein-like 4 | 84871 | 37.99 | 0.31 | 0.73 | 0.81 |
| IGF-like family member 4 | 444882 | 24.45 | 0.31 | 0.76 | 0.70 |
| serine peptidase inhibitor, Kunitz type, 2 | 10653 | 28.72 | 0.31 | 0.70 | 0.75 |
| brain and reproductive organ-expressed (TNFRSF1A modulator) | 9577 | 40.51 | 0.31 | 0.77 | 0.86 |
| microtubule-associated protein, RP/EB family, member 3 | 22924 | 0.00 | 0.30 | 0.74 | 0.89 |
| uncharacterized LOC100288315 | 1E+08 | 27.36 | 0.30 | 0.73 | 0.78 |
| glyoxalase domain containing 4 | 51031 | 35.91 | 0.30 | 0.74 | 0.81 |
| Rho GDP dissociation inhibitor (GDI) alpha | 396 | 13.79 | 0.30 | 0.71 | 0.72 |
| RAS p21 protein activator (GTPase activating protein) 1 | 5921 | 23.21 | 0.30 | 0.75 | 0.90 |
| solute carrier family 36 (proton/amino acid symporter), member 1 | 206358 | 13.09 | 0.29 | 0.72 | 0.81 |
| N(alpha)-acetyltransferase 30, NatC catalytic subunit | 122830 | 18.10 | 0.28 | 0.70 | 0.87 |
| CAP-GLY domain containing linker protein 3 | 25999 | 8.55 | 0.28 | 0.73 | 0.86 |
| nuclear pore complex-interacting protein-like 1-like | 1E+08 | 12.92 | 0.28 | 0.72 | 0.77 |
| nuclear pore complex-interacting protein-like 1-like | 642778 | 11.86 | 0.28 | 0.73 | 0.87 |
| epidermal growth factor | 1950 | 0.21 | 0.28 | 0.74 | 0.74 |
| nerve growth factor receptor (TNFRSF16) associated protein 1 | 27018 | 34.55 | 0.28 | 0.70 | 0.83 |
| AT rich interactive domain 4A (RBP1-like) | 5926 | 41.71 | 0.27 | 0.72 | 0.83 |
| WD repeat domain 47 | 22911 | 46.32 | 0.27 | 0.71 | 0.70 |
| ring finger protein 148 | 378925 | 33.74 | 0.26 | 0.74 | 0.83 |
| HIG1 hypoxia inducible domain family, member 2B | 123346 | 18.77 | 0.26 | 0.76 | 0.90 |
| F-box protein 21 | 23014 | 22.07 | 0.26 | 0.75 | 0.91 |
| SH2 domain containing 1B | 117157 | 27.96 | 0.26 | 0.75 | 0.66 |
| sprouty-related, EVH1 domain containing 2 | 200734 | 29.80 | 0.26 | 0.70 | 0.72 |
| lin-7 homolog C (C. elegans) | 55327 | 29.28 | 0.25 | 0.72 | 0.88 |
| tectonin beta-propeller repeat containing 1 | 25851 | 28.44 | 0.25 | 0.72 | 0.83 |
| ubiquilin 2 | 29978 | 44.12 | 0.25 | 0.71 | 0.69 |
| chromosome 19 open reading frame 12 | 83636 | 45.16 | 0.25 | 0.73 | 0.73 |
| adaptor-related protein complex 3, mu 2 subunit | 10947 | 20.62 | 0.24 | 0.71 | 0.86 |
| DENN/MADD domain containing 1A | 57706 | 22.37 | 0.24 | 0.71 | 0.75 |
| ring finger protein 11 | 26994 | 13.10 | 0.24 | 0.70 | 0.87 |
| ganglioside induced differentiation associated protein 1 | 54332 | 26.46 | 0.23 | 0.72 | 0.85 |
| RAB GTPase activating protein 1 | 23637 | 27.50 | 0.23 | 0.72 | 0.84 |
| ubiquitin specific peptidase 10 | 9100 | 25.57 | 0.23 | 0.73 | 0.89 |
| MAX binding protein | 4335 | 28.05 | 0.22 | 0.74 | 0.78 |
| armadillo repeat containing, X-linked 2 | 9823 | 19.61 | 0.22 | 0.73 | 0.83 |
| leucine rich repeat containing 27 | 80313 | 40.35 | 0.22 | 0.70 | 0.74 |
| zinc finger protein 346 | 23567 | 9.35 | 0.22 | 0.73 | 0.82 |
| coiled-coil domain containing 112 | 153733 | 57.75 | 0.22 | 0.71 | 0.85 |
| GLIS family zinc finger 1 | 148979 | 7.07 | 0.22 | 0.73 | 0.67 |
| IQ motif and Sec7 domain 3 | 440073 | 15.61 | 0.22 | 0.71 | 0.69 |
| CDKN2A interacting protein N-terminal like | 91368 | 15.99 | 0.22 | 0.76 | 0.82 |
| acyl-CoA thioesterase 4 pseudogene | 644189 | 23.53 | 0.21 | 0.78 | 0.82 |
| KIAA0947 | 23379 | 32.45 | 0.21 | 0.74 | 0.87 |
| tRNA nucleotidyl transferase, CCA-adding, 1 | 51095 | 41.02 | 0.21 | 0.75 | 0.91 |
| molybdenum cofactor synthesis 3 | 27304 | 21.58 | 0.21 | 0.73 | 0.86 |
| chromosome 1 open reading frame 21 | 81563 | 32.33 | 0.20 | 0.76 | 0.84 |
| cell adhesion molecule 3 | 57863 | 28.67 | 0.20 | 0.70 | 0.72 |
| ATPase, H+ transporting, lysosomal 34kDa, V1 subunit D | 51382 | 14.60 | 0.20 | 0.77 | 0.90 |
| solute carrier family 39 (zinc transporter), member 10 | 57181 | 16.52 | 0.20 | 0.72 | 0.72 |
| transmembrane protein 181 | 57583 | 22.25 | 0.20 | 0.70 | 0.74 |
| G patch domain containing 2 | 55105 | 27.06 | 0.20 | 0.74 | 0.88 |
| ubiquitin specific peptidase 46 | 64854 | 47.04 | 0.20 | 0.71 | 0.77 |
| DnaJ (Hsp40) homolog, subfamily C, member 5 | 80331 | 7.43 | 0.20 | 0.74 | 0.85 |
| WD repeat domain 5 | 11091 | 15.81 | 0.20 | 0.76 | 0.87 |
| eukaryotic translation initiation factor 3, subunit C | 8663 | 27.85 | 0.20 | 0.72 | 0.80 |
| low density lipoprotein receptor-related protein 11 | 84918 | 16.40 | 0.19 | 0.77 | 0.79 |
| Sjogren syndrome/scleroderma autoantigen 1 | 10534 | 19.12 | 0.19 | 0.73 | 0.84 |
| zinc finger, RAN-binding domain containing 3 | 84083 | 42.31 | 0.19 | 0.71 | 0.82 |
| RAS p21 protein activator 2 | 5922 | 13.39 | 0.19 | 0.73 | 0.85 |
| protein kinase, AMP-activated, alpha 2 catalytic subunit | 5563 | 16.09 | 0.19 | 0.72 | 0.72 |
| suppression of tumorigenicity 7 | 7982 | 11.49 | 0.18 | 0.73 | 0.88 |
| adiponectin receptor 1 | 51094 | 5.49 | 0.18 | 0.71 | 0.78 |
| POM121 and ZP3 fusion | 22932 | 19.34 | 0.18 | 0.72 | 0.83 |
| family with sequence similarity 175, member B | 23172 | 12.13 | 0.17 | 0.72 | 0.87 |
| chromosome 14 open reading frame 169 | 79697 | 30.47 | 0.17 | 0.73 | 0.86 |
| hypoxia up-regulated 1 | 10525 | 0.00 | 0.16 | 0.72 | 0.81 |
| M-phase phosphoprotein 6 | 10200 | 17.24 | 0.16 | 0.71 | 0.84 |
| RAB2A, member RAS oncogene family | 5862 | 19.25 | 0.16 | 0.71 | 0.83 |
| dynein, light chain, LC8-type 1 | 8655 | 7.69 | 0.16 | 0.70 | 0.85 |
| microtubule-associated protein 6 | 4135 | 7.56 | 0.15 | 0.72 | 0.85 |
| oxysterol binding protein | 5007 | 0.00 | 0.15 | 0.73 | 0.81 |
| dopamine beta-hydroxylase (dopamine beta-monooxygenase) | 1621 | 0.00 | 0.14 | 0.71 | 0.69 |
| nitrogen permease regulator-like 3 (S. cerevisiae) | 8131 | 18.23 | 0.14 | 0.71 | 0.80 |
| homeobox A6 | 3203 | 14.00 | 0.13 | 0.71 | 0.80 |
